# Association Between Serum Ustekinumab Concentrations and Endoscopic Disease Activity in Moderate-to-Severe Crohn’s Disease Patients

**DOI:** 10.1093/crocol/otae071

**Published:** 2024-11-29

**Authors:** David M P Di Fonzo, Balqis Alabdulkarim, Russell Yanofsky, Yaqeen Abduallah, Petra Golovics, Peter L Lakatos, Alain Bitton, Gary Wild, Waqqas Afif, Talat Bessissow

**Affiliations:** Department of Medicine, McGill University Health Centre, Montreal, QC, Canada; Department of Medicine, McGill University Health Centre, Montreal, QC, Canada; Division of Gastroenterology and Hepatology, University of Toronto, Toronto, ON, Canada; Department of Medicine, McGill University Health Centre, Montreal, QC, Canada; Division of Gastroenterology and Hepatology, McGill University Health Centre, Montreal, QC, Canada; Department of Gastroenterology, Central Hospital of Northern Pest– Military Hospital, Budapest, Hungary; Division of Gastroenterology and Hepatology, McGill University Health Centre, Montreal, QC, Canada; Department of Internal Medicine and Oncology, Semmelweis University, Budapest, Hungary; Division of Gastroenterology and Hepatology, McGill University Health Centre, Montreal, QC, Canada; Division of Gastroenterology and Hepatology, McGill University Health Centre, Montreal, QC, Canada; Division of Gastroenterology and Hepatology, McGill University Health Centre, Montreal, QC, Canada; Division of Gastroenterology and Hepatology, McGill University Health Centre, Montreal, QC, Canada

**Keywords:** biologic, Crohn’s disease, endoscopy

## Abstract

**Background/Aims:**

The role of ustekinumab therapeutic drug monitoring in patients with Crohn’s disease (CD) remains ambiguous. Examination of the association serum ustekinumab concentrations and endoscopic outcomes has yielded inconsistent results. Our study examined whether serum ustekinumab concentrations were associated with endoscopic healing in patients with moderate-to-severe CD.

**Methods:**

This was a cross-sectional study of adult patients with CD on maintenance ustekinumab. Patients were included if they had serum ustekinumab concentrations and endoscopic evaluation taken within 4 months of each other. Endoscopic healing was defined as absence of ulceration on endoscopy or Simplified Endoscopic Score for Crohn’s disease (SES-CD) < 3. Quartile analysis of drug levels was performed, and receiver operating characteristic curve was calculated. Multivariate logistic regression assessed for the probability of endoscopic healing based on serum ustekinumab concentration.

**Results:**

Seventy-four patients were included in the final analysis. The mean serum ustekinumab concentration of the population was 6.10 mcg/mL. Serum ustekinumab concentration did not predict endoscopic remission based on either the absence of ulceration or SES-CD < 3. There was no difference in the frequency of ulceration at increasing serum ustekinumab concentrations. There was no threshold serum ustekinumab concentration associated with the absence of ulceration (area under the curve [AUC] = 0.50) or SES-CD < 3 (AUC = 0.49).

**Conclusions:**

Our study found no association between serum ustekinumab concentrations and endoscopic remission in patients with CD. Exploration of mechanisms accounting for this lack of association is warranted.

## Graphical Abstract

**Graphical Abstract:**
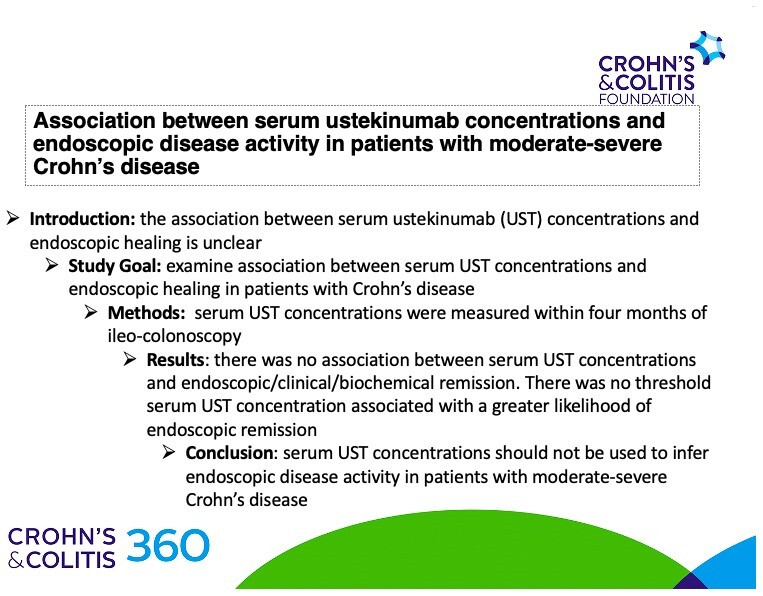

## Introduction

Crohn’s disease (CD) continues to rise in global incidence, posing a significant morbidity burden to affected populations.^1^ Based on the recent recommendations of the Selecting Therapeutic Targets in Inflammatory Bowel Disease (STRIDE-II) program, important CD treatment endpoints include clinical and endoscopic remission.^2^ Endoscopic remission, defined by STRIDE-II as a simple endoscopic score < 3 and lack of ulceration,^2^ is especially relevant as this outcome has been shown to be associated with a greater likelihood of steroid-free remission and reduced risk of relapse, hospitalization, and surgery.^3–5^

Biologic agents are often used in the management of moderate-to-severe cases of CD as they have demonstrated efficacy in improving patient symptomology and reducing endoscopic inflammation.^6^ Ustekinumab (UST) (Stelara; Janssen Biotech, Inc., Horsham, PA), a monoclonal antibody, is a biological agent frequently employed in the treatment of moderate-severe CD, which has failed previous biologic therapy.^6^ In the UNITI-1, UNITI-2, and IM-UNITI trials, UST was shown to be effective in inducing and maintaining clinical and biochemical remission in patients with CD.^7,8^ Given the increased usage of this agent, means of guiding titration of UST are currently under investigation, and among the potential options is therapeutic drug monitoring (TDM). TDM is the practice of measuring drugs at certain intervals to maintain a stable concentration in a patient’s serum, with the intention of optimizing drug delivery.^9^ TDM has been previously shown to have an impact on treatment decisions for CD patients treated with antitumor necrosis factor alpha (TNF-alpha) inhibitors.^10^

When examining the role of TDM in UST, the UNITI-1, UNITI-2, and IM-UNITI trials each found a positive association between serum UST trough levels and clinical remission in CD.^7,8^ Similarly, in the UNIFI study, Adedokun et al. (2020) used quartile analysis to demonstrate that a positive exposure–response relationship existed with higher serum UST levels associated with higher rates of clinical and endoscopic response in patients with ulcerative colitis.^11^ In addition, a threshold of 3.7 mcg/mL at week 8 was identified as a target for achieving clinical response (AUC 0.65; 95% CI, 0.61-0.69; *P* < .001).^11^ There is also evidence that serum concentrations of UST are associated with evidence of histological remission.^12^ However, the association between serum UST concentrations and endoscopic outcomes is less straightforward. For instance, when examining endoscopic remission based on quartile analysis of UST TDM, Adedoukun et al. (2018) found that there was no significant increase in endoscopic remission rates at week 8 despite a 2-fold increase in serum UST concentration after induction.^13^ However, when examining endoscopic remission in the maintenance phase, the frequency of endoscopic remission increased between the second and fourth quartile of serum UST concentration.^13^ Moreover, prior studies that have demonstrated a positive association between serum UST concentrations and endoscopic healing have diverged as to what therapeutic drug level is necessary to achieve endoscopic healing. In one study, there was a significant negative correlation between serum UST concentration and the presence of endoscopic CD activity; however, area under the curve (AUC) analysis was unable to identify a target threshold value for endoscopic response.^14^ In other studies, different serum UST concentration thresholds were demonstrated to correlate with endoscopic healing.^15–20^ The precise therapeutic drug level to target has differed between studies. For instance, Battat and colleagues (2017) identified 4.5 mcg/mL as a target 26-week trough threshold above which 76% of patients experienced an endoscopic response compared with 41% of those who did not achieve this target level (*P* = .08). While not significant, there was also a trend toward higher endoscopic remission rates in patients with a 26-week tough level > 4.5 mcg/mL (*P* = .1).^15^ Interestingly, there was no target trough level identified at which clinical response or clinical remission was more likely.^15^ In partial concordance, Verstockt et al. (2019) found that serum UST levels below 5.0 mcg/mL at week 8 had a negative predictive value (NPV) of 90% for future endoscopic response though this finding was not significant (*P* = .25).^16^ Furthermore, the authors identified a threshold of 2.3 mcg/mL at week 16 as the minimal threshold to maximize the likelihood of endoscopic response at 6 months (NPV 88%, *P* = .10), while at week 24, the level was 1.9 mcg/mL (NPV 85%; *P* = .22).^16^ Walshe and colleagues (2021) identified 3.75 mcg/mL as the target threshold to achieve endoscopic remission in patients with CD (AUC 0.725; 95% CI, 0.644-0.805; *P* < .001).^17^ Yao et al. (2021) identified a lower threshold level as they found that week 16 trough levels of UST above 1.12 mcg/mL predicted a higher likelihood of endoscopic remission (AUC 0.78, sensitivity 88%, specificity 73%).^18^ In lieu of such findings, there is currently no clear consensus as to what UST threshold level, if any, is reasonable to target in order to optimize the rates of endoscopic or clinical remission in patients with CD. Owing to such discrepancies in findings, the use of ustekinumab TDM is currently not standard of care.^6^

Given the unresolved nature of the association between serum UST concentration and endoscopic healing in CD, it is important to further elucidate if an association between these 2 variables exists and what is the precise nature of the interaction. Therefore, the purpose of this study was to examine the association between serum UST concentration and the presence of endoscopic healing in patients with CD. Additionally, this study aimed to determine a serum UST concentration threshold associated with endoscopic healing in this cohort.

## Materials and Methods

### Patients

We assessed adult patients with moderate-to-severe CD, undergoing maintenance treatment with ustekinumab, who were undergoing surveillance ileo-colonoscopy to assess disease activity. We excluded patients who were pregnant or with an ileal-pouch anal anastomosis. The patients were recruited at the McGill University Health Center which consists of 2 hospitals: Montreal General Hospital and Royal Victoria Hospital (both situated in Montreal, Canada). The recruitment period was extended from January 2018 to December 2022.

### Study Design

We performed a cross-sectional study, with data collected from patients at the time of surveillance ileo-colonoscopy. The morning before endoscopy, prior to initiating bowel preparation, the patients collected a stool sample for fecal calprotectin (FC). Serum C-reactive protein (CRP) levels were also measured on the day of endoscopy. Random serum UST concentrations were measured within 4 months of the index colonoscopy using an enzyme-linked immunosorbent assay (ELISA) (Dynacare Laboratories Inc., Brampton, ON). Endoscopic evaluations occurred using standard endoscopic preparation and techniques. A simplified endoscopic score for Crohn’s disease (SES-CD) was calculated either at the time of endoscopy by a trained gastroenterologist or upon review of endoscopy reports and images using trained research assistants.

### Study Variables

At the time of study recruitment, the patient’s demographic and clinical data were collected. This included the patient’s age at the time of study inclusion, sex, date of CD diagnosis (year of diagnosis), smoking status (current smoker, ex-smoker, or never-smoker), and Montreal classification of CD (extent of disease involvement and phenotype of disease). Use of current immunosuppressive (methotrexate, azathioprine, or 6-mercatopurine) and previous medical therapy (5-ASA, methotrexate, azathioprine, 6-mercatopurine, anti-TNF alpha therapy, and anti-integrin therapy) were also recorded. UST doses (induction and maintenance dosing) were recorded at the time of recruitment. Data on previous intestinal surgical resections were also collected. The clinical activity of CD was assessed using the Harvey Bradshaw Index (HBI) (HBI < 5 corresponding to clinical remission). Using retrospective chart review of electronic medical records, clinical (HBI), biomarker (CRP/FC), and endoscopic data were retrieved at the time of initial endoscopy in relation to the study (prior to initiation of UST therapy) for patients included in analysis. We also recorded whether a patient received standard UST induction therapy and the start date of UST therapy to determine the duration of treatment.

### Endpoints and Definitions

The primary endpoint was to assess whether serum UST concentration was significantly associated with endoscopic remission, defined as the absence of ulceration (as described by the endoscopist) or endoscopic remission (SES-CD < 3). Secondary endpoints examined whether serum UST concentration was associated with clinically quiescent disease (HBI score < 5) and biomarker remission (CRP < 5 mg/L or FC < 200 mcg/g). Deep remission was calculated using a composite score consisting of clinical (HBI < 5), biochemical (FC < 200 + CRP < 5), and endoscopic (absence of ulceration) remission. Patient subgroups were created stratifying patients based on the following features: prior anti-TNF use, intensification of UST dosing (defined as Q4 week/Q2 week dosing), deep remission, and TDM taken within 1 month of surveillance endoscopy.

### Statistical Analysis

Data were inspected to ensure whether the assumptions of normal distribution were met. Descriptive statistics were calculated for continuous (mean ± SD) and categorical variables (percentage). Receiver operating characteristic (ROC) curve and area under curve (AUC) were modeled using test sensitivity and specificity to determine a serum UST concentration threshold associated with endoscopic healing. Quartile analysis was performed to assess whether endoscopic healing rates differed at increasing levels of serum UST concentration. For main analyses, logistic regression was performed using univariate and multivariate analysis to examine associations between serum UST concentration and endoscopic remission. Logistic regression was repeated to examine associations between serum UST concentration and endoscopic remission in subgroup stratification. All multivariate models (in both main and subgroup analyses) were adjusted for age at inclusion, sex, and height in centimeters. Significance was set at *P* ≤ .05. Statistical analyses were performed using SAS software version 3.8 (SAS Institute, Cary, NC).

### Ethics

The protocol and materials of the current study received ethical approval by the research ethics board at our institution (Ethics No. 2019-5060). Patients provided informed consent for inclusion in the current study. All study authors reviewed and approved the manuscript before submission.

## Results

### Baseline Patients Characteristics

Seventy-four patients were included in the final study analysis. Patient characteristics (*n* = 74) are stratified based on the presence/absence of ulceration on endoscopy (Table 1). Mean age at diagnosis was 25.97 ± 11.26 years in those with ulceration on endoscopy and 28.50 ± 11.95 years in those without ulceration on endoscopy (Table 1). The mean age of patients at the time of inclusion was 40.3 years. At the time of study inclusion, 35% of patients had prior CD-related surgery (*n* = 26), 24% had prior or active peri-anal disease (*n* = 18) and 62 patients had previously failed anti-TNF alpha therapy, and 5% of the patients were on concurrent immunosuppressive therapy (*n* = 4) and 9% were on concomitant steroid therapy (*n* = 7). At the time of TDM, 34% (*n* = 25) of patients were in endoscopic remission based on SES-CD < 3, while 49% (*n* = 36) of patients were in endoscopic remission based on no ulceration present on endoscopy, and 16% (*n* = 12) of the patients had achieved deep remission. Samples differed based on disease behavior as patients with no ulceration on endoscopy were less likely to have penetrating/structuring disease (*P* = .03; Table 1). Additionally, patients with no ulceration were more likely to have less severe disease on biopsy (*P* = .001; Table 1), less likely to have had prior inflammatory bowel disease (IBD)-related surgery (*P* = .02; Table 1) and had lower FC (*P* = .003; Table 1), and 46% (*n* = 34) of the population had proactive TDM (TDM occurring within 4 months before endoscopy) and 54% (*n* = 40) had reactive TDM (TDM occurring within 4 months after endoscopy); 97% (*n* = 72) of the patient population had TDM within 3 months before/after endoscopic assessment, and 86% (*n* = 64) of the patient population had TDM measured within 1 month before/after endoscopic assessment. We did not record which patients were on intensified dosing (Q2/Q4 week dosing) and whether they had their dose intensification before colonoscopy. No significant difference in serum UST concentration was found when comparing the sample based on endoscopic remission (SES CD < 3 and absence of ulceration), clinical remission, normal FC, and deep remission (Table 2). Interestingly, patients with a normal CRP (<5 mg/L) had a significantly higher serum UST concentrations (*P* = .04) (Table 2).

**Table 1.** Patient population characteristics stratified based on the presence of ulceration.

	Ulcer present *N* = 38		Ulcer absent *N* = 36		*P*-value
Age at inclusion (years), Mean (SD)	40.63 (14.23)		39.94 (14.09)		.77
Age at diagnosis (years), Mean (SD)	25.97 (11.26)		28.50 (11.95)		.45
Weight (kg), Mean (SD)	72.17 (16.23)		78.32 (25.52)		.51
Height (cm), Mean (SD)	167.76 (8.45)		166.59 (10.41)		.74
Serum UST Concentration (mcg/ml), Mean (SD)	6.50 (5.53)		5.69 (4.62)		.66
CRP (mg/L), Mean (SD)	19.22 (26.37)		15.95 (18.34)		.54
FC (mcg/g), Mean (SD)	846.20 (1196.97)		638.59 (1805.73)		.0039^b^
HBI, Mean (SD)	7.36 (3.99)		9.77 (8.48)		.45
Sex (%)					
Male	21 (55.3)		16 (44.4)		.35
Female	17 (44.7)		20 (55.6)		
Behavior (%)					
B1	20 (52.6)		27 (77.1)		.03^a,b^
B2	12 (31.6)		3 (8.6)		
B3	6 (15.8)		5 (14.3)		
Location (%)					
L1	6 (15.8)		4 (11.4)		.20^a^
L2	11 (28.9)		17 (48.6)		
L3	19 (50.0)		13 (37.1)		
L4	2 (5.3)		1 (2.9)		
Biopsy (%)					
Inactive	4 (11.4)		16 (53.3)		.0012^b^
Mild	16 (45.7)		8 (26.7)		
Moderate	12 (34.3)		3 (10.0)		
Severe	3 (8.6)		3 (10.0)		
Prior Anti-TNF Exposure (%)					
Yes	33 (91.7)		29 (80.6)		.31
No	3 (8.3)		7 (19.4)		
Perianal disease history (%)					
Never	28 (73.7)		27 (77.1)		.43^a^
Prior history	8 (21.1)		4 (11.4)		
Currently active	2 (5.3)		4 (11.4)		
Prior IBD-related surgery (%)					
Yes	18 (47.4)		8 (22.2)		.02^b^
No	20 (52.6)		28 (77.8)		
Concomitant steroid use (%)					
Yes	6 (15.8)		1 (2.8)		.11^a^
No	32 (84.2)		35 (97.2)		
Concomitant immunosuppressant use (%)					
Yes	3 (7.9)		1 (2.8)		.62^a^
No	35 (92.1)		35 (97.2)		
Ustekinumab maintenance dose frequency (%)					
Q2 weeks	2 (5.3)		0 (0.0)		.06^a^
Q4 weeks	23 (60.5)		15 (41.7)		
Q6 weeks	0 (0.0)		1 (2.8)		
Q8 weeks	13 (34.2)		20 (55.6)		
	Ulcer present (*N* = 38)		Ulcer absent (*N* = 36)		*P*-value
	Median	IQR	Median	IQR	
Age at diagnosis (years)	24.00	11.00	27.50	19.50	.45
Age at inclusion (years)	39.50	27.00	38.00	20.00	.77
Weight (kg)	69.00	20.50	67.00	23.00	.51
Height (cm)	170.00	13.00	167.00	17.00	.74
TDM (mcg/mL)	5.04	7.30	3.91	5.47	.66
CRP (mg/L)	9.20	14.86	7.23	28.90	.54
FC (mcg/g)	355.50	568.00	107.50	224.00	.0039^b^
HBI	8.00	4.50	8.00	5.00	.45

^a^Denotes use of Fisher Exact Test.

^b^Denotes statistical significance.

**Table 2.** Comparison of mean serum UST concentration using subgroup stratification.

Outcome	Yes			No			
	*N*	Mean	SD	*N*	Mean	SD	*P*-value
**Endoscopic remission (SES CD < 3)**	25	5.57	4.26	49	6.37	5.48	.8059
**Endoscopic remission (no ulceration)**	36	5.69	4.62	38	6.50	5.53	.6653
**Clinical remission (HBI < 4)**	45	6.06	4.77	24	6.56	6.03	.8157
**Normal CRP (<5 mg/L)**	48	6.85	5.26	24	4.69	4.69	.0460^a^
**Normal FC (<200 mcg/g)**	32	5.94	4.47	30	6.14	5.38	.8107
**Deep remission**	12	7.07	6.22	59	5.97	4.99	.7127

^a^Denotes statistical significance.

### Ustekinumab TDM and Endoscopic Remission

**Figure 1.**
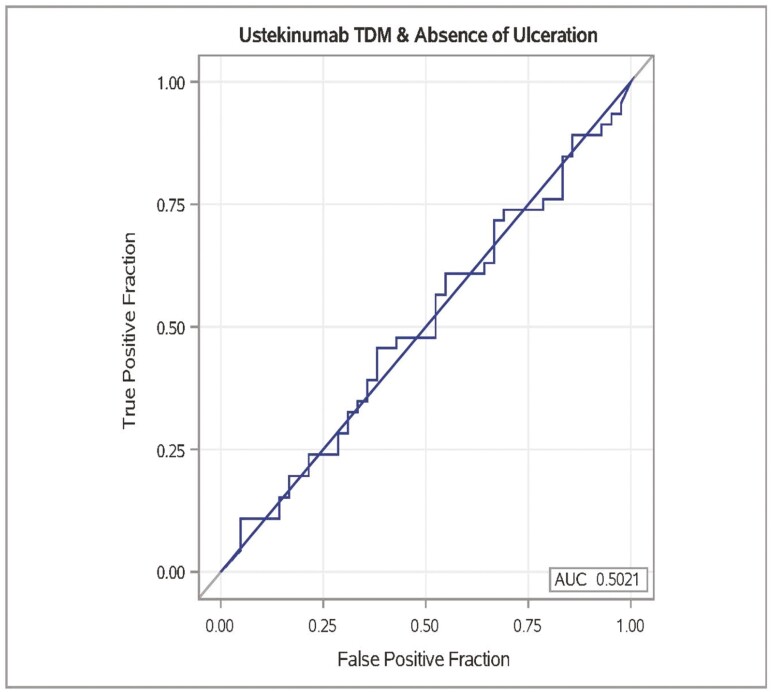
ROC analysis for the presence of endoscopic remission (no ulceration on endoscopy) based on serum UST concentration. Best receiver-operating characteristic curve (area under the curve, 0.50; sensitivity, 14%; specificity, 89%).

**Figure 2.**
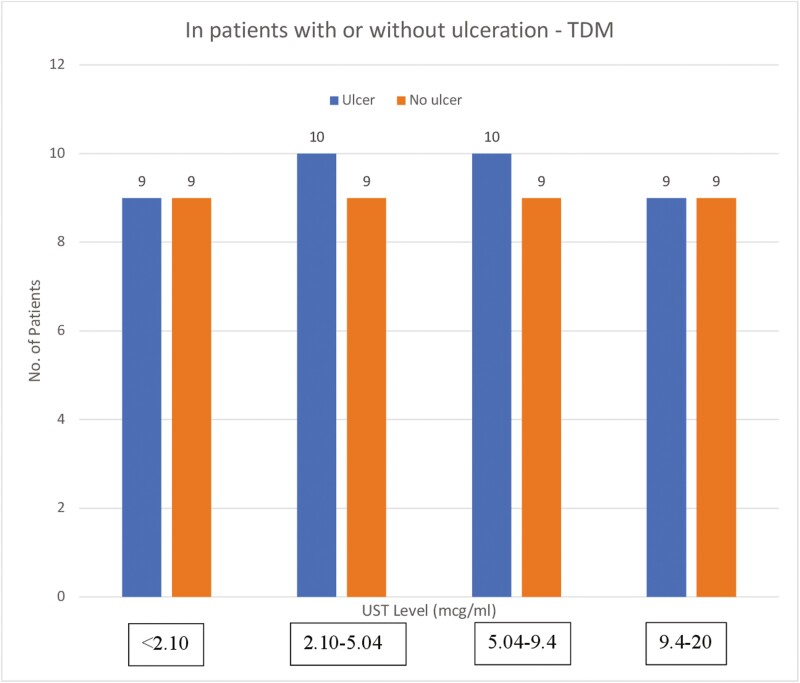
Quartile analysis of evidence of ulceration based on serum UST concentration.

**Figure 3.**
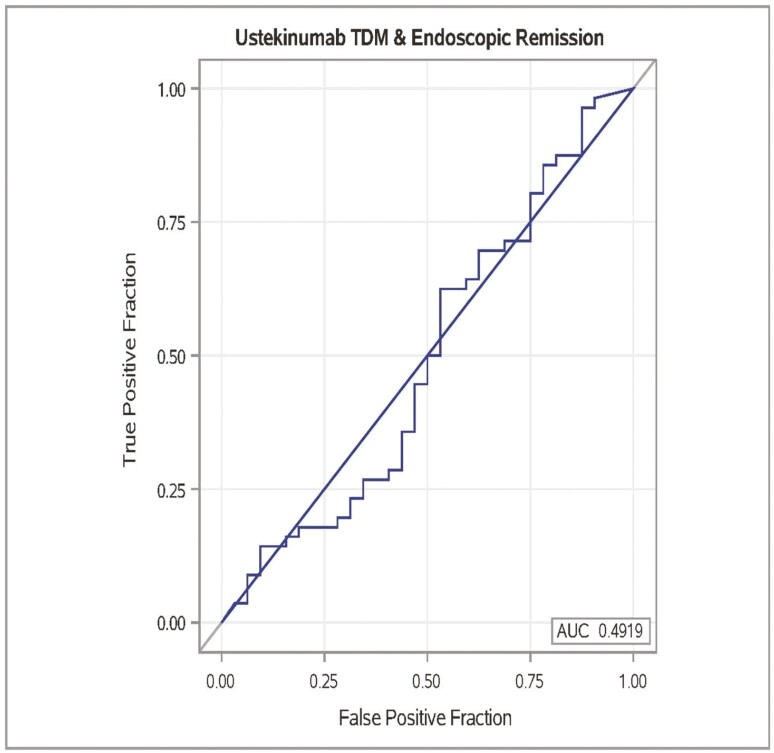
ROC curve analysis for endoscopic remission (SES-CD < 3) based on serum UST concentration. Best receiver-operating characteristic curve (area under the curve, 0.49; sensitivity, 22%; specificity, 86%).

At the time of endoscopy, mean serum UST concentration was 6.50 ± 5.53 mcg/mL in the patients with ulceration (Table 1) and 5.69 ± 4.62 mcg/mL in patients with no ulceration (Table 1). When applying multivariate logistic regression, serum UST concentration did not significantly predict the absence of ulceration on endoscopy (Table 3; Multivariate OR 0.99; CI, 0.90-1.08; *P* = .79). ROC analysis yielded no serum UST concentration threshold associated with the absence of ulceration on endoscopy (Figure 1; AUC = 0.50; sensitivity, 14%; specificity, 89%). Quartile analysis did not demonstrate any significant difference between the absence of ulceration based on serum UST concentrations divided into quartiles (Figure 2). Similarly, when applying multivariate logistic regression, serum UST concentration did not predict endoscopic remission (SES-CD < 3) on endoscopy (Table 3; OR 1.04; CI, 0.95-1.15; *P* = .38). ROC analysis yielded no serum UST concentration threshold associated with endoscopic remission (SES-CD < 3) on endoscopy (Figure 3; AUC = 0.49; sensitivity, 22%; specificity, 86%).

**Table 3.** Results of univariate and multivariate logistic regression examining association between serum UST concentration and endoscopic, clinical and biochemical remission.

Outcome	Univariate			Multivariate		
	Odds ratio	95% CI	*P*-value	Odds ratio^a^	95% CI	*P*-value
**Endoscopic remission (SES-CD < 3)**	1.02	(0.94-1.11)	.6431	1.04	(0.95-1.15)	.3824
**Endoscopic remission (no ulceration)**	1.01	(0.93-1.09)	.8785	0.99	(0.90-1.08)	.7945
**Clinical remission (HBI < 5)**	0.97	(0.89-1.06)	.5009	0.97	(0.87-1.09)	.6407
**CRP (<5 mg/L)**	1.05	(0.96-1.16)	.2953	-	-	-
**FC (<200 mcg/g)**	1.00	(0.87-1.15)	.9854	1.02	(0.84-1.22)	.8690

^a^Multivariate models are all adjusted for age of inclusion, sex, and height.

### Ustekinumab TDM and Clinical/Biochemical Outcomes

When applying univariate logistic regression, serum UST concentration did not significantly predict serum levels of CRP (Table 3; OR 1.05; CI, 0.96-1.16; *P* = .30). When applying multivariate logistic regression, serum UST concentration did not significantly predict the levels of FC (Table 3; OR 1.02; CI, 0.87-1.15; *P* = .99). When applying multivariate logistic regression, serum UST concentration did not significantly predict clinically inactive disease (HBI < 5) (Table 3; OR 0.97, CI, 0.87-1.09; *P* = .64).

### Subgroup Analyses

Results from the subgroup analysis demonstrated that the lack of association between serum UST concentration and endoscopic remission (absence of ulceration) did not differ based on multiple different factors. Specifically, when applying univariate logistic regression, serum UST concentration did not predict endoscopic remission (absence of ulceration) on endoscopy when stratifying patients based on the following characteristics: prior anti-TNF exposure compared with no prior anti-TNF exposure (Table 4; OR 0.89; CI, 0.67-1.18; *P* = .41), intensified UST dosing versus nonintensified UST dosing (Table 4; OR 0.94; CI, 0.83-1.06; *P* = .32), evidence of deep remission versus no evidence of deep remission (Table 4; OR 0.94; CI, 0.83-1.06; *P* = .28), or patients with TDM within 1 month of endoscopy versus TDM > 1 month from endoscopy (Table 4; OR 0.97; CI, 0.88-1.06; *P* = .48).

**Table 4.** Results of univariate and multivariate logistic regression, using subgroup stratification, examining association between serum UST concentration and endoscopic remission (no ulceration).

Outcome	Univariate			Multivariate		
	Odds ratio	95% CI	*P*-value	Odds ratio^a^	95% CI	*P*-value
**Prior anti-TNF exposure**	0.89	(0.67-1.18)	.4084	0.79		.9818
**Intensified UST dosing**	0.94	(0.83-1.06)	.3192	0.91	(0.79-1.06)	.2219
**Deep remission**	0.94	(0.83-1.06)	.2801			
**TDM within 1 month of endoscopy**	0.97	(0.88-1.06)	.4814	0.98	(0.88-1.09)	.7518

^a^Multivariate models are all adjusted for age of inclusion, sex, and height.

## Discussion

Our study demonstrated that serum UST concentrations were not associated with endoscopic healing assessed via 2 important metrics: SES-CD < 3 and the absence of ulceration.^2^ Few studies have concurrently examined both markers of endoscopic healing, and therefore, our study offers an especially robust assessment of this outcome.

Approximately one-third of IBD patients will lose endoscopic response to biologic agents.^21^ As such, TDM may assist practitioners in both preventing and addressing loss of response to therapy.^22^ Despite demonstrated efficacy in anti-TNF agents,^10^ the utility of UST TDM remains a controversial subject. Studies that have demonstrated an association between serum UST concentration and endoscopic healing have been unable to identify a consistent target threshold value.^14–20^ When examining our semi-discordant findings in lieu of previous research, multiple challenges emerge when attempting to draw parallels between studies.

One major challenge in examining the role of TDM lies in the variability with which endoscopic healing is defined. In a cross-sectional analysis of 28 patients with CD, Hirayama et al. (2022) found that serum UST levels were significantly higher in patients with endoscopic remission as defined by an adapted Rutgeerts score of 0 (no lesions) or 1 (≤5 aphthous lesions).^14^ However, endoscopic remission was only achieved in 8 patients.^14^ In the current study, we had 37 patients who met the strict criteria of no ulceration on endoscopy and labeled this cohort as endoscopic remission. As such, through using a more stringent definition of endoscopic remission this may have accounted for our unique findings.

Another challenge lies in the timing of UST TDM, as target therapeutic drug concentration cutoffs may differ based on whether TDM occurred in the induction or maintenance phase.^23^ Moreover, even when TDM occurs in the same phase, trough concentrations will often differ from concentrations which are taken between administrations.^23^ For example, Hanzel et al. (2020) demonstrated that peak serum ustekinumab levels at weeks 2, 4, and 8 (after induction therapy) predicted endoscopic remission (SES-CD < 3) at 24 weeks. Furthermore, Walshe et al. (2021) demonstrated that higher serum UST levels, sampled within 1 month of endoscopic assessment, were associated with a higher number of subjects in endoscopic remission as assessed by endoscopic healing index (EHI < 20). In this cohort, however, the authors were unable to confirm the timing of TDM in relation to dose administration. Conversely, Battat et al. (2017) found that ustekinumab levels ≥4.5 ug/mL at week 26 of treatment were associated with higher rates of endoscopic response but not endoscopic remission. In addition, serum UST concentrations measured at week 10 of therapy did not yield a threshold level associated with endoscopic response/remission at either week 10 or 26. Furthermore, Painchart et al. (2020) found no significant difference in endoscopic response to treatment when comparing median serum UST trough levels at week 12 of treatment. In the current study, we performed TDM in patients, while in the maintenance phase of treatment, therefore, it is difficult to compare across studies that have measured UST concentrations at other times. As the interplay between UST TDM and endoscopic response may vary over time, a comparison of TDM taken at identical time periods is necessary to draw proper comparisons.

Another important consideration is the notion that the use of different assays has been known to cause difficulties when comparing results across studies.^23^ Our study employed an ELISA to measure serum UST concentration. Previous studies have used either ELISA or HMSA.^14–20^ Importantly, Verdon et al. demonstrated that serum UST concentrations obtained via Prometheus HMSA correlated poorly with ELISA, as the levels were approximately twice as high in the HMSA version. This discordance in measurement may also partially account for the lack of consistency across trials when comparing the utility of TDM.

Finally, it is important to note that serum drug levels do not necessarily correlate with drug receptor activity at the level of the colonic mucosa.^23^ When comparing anti-TNF levels in the serum and tissue, Yarur et al. (2015) found that there was a significant correlation between these values for patients on infliximab but not adalimumab. The correlation between serum and tissue drug levels was significant when tissues were uninflamed but failed to reach significance in inflamed tissue.^24^ Patients who are nonresponsive to anti-TNF therapy tend to have higher serum to tissue ratio of concentration, suggesting that tissue concentration may be the more predictive factor of response to therapy.^25^ In the current study, only 36% of the patients were in endoscopic remission; therefore, many patients had significant inflammation which may account for the lack of an association between these variables.

Overall, we acknowledge that the results of the current study differ from prior research into the association between UST TDM and endoscopic healing, specifically the aforementioned UNITI Trials.^7^ As previously discussed, the use of a different assay for TDM may introduce variability into study results^23^ and it is possible that this may have led to discordance between our trial and the UNITI trials where the exact assay used was not described. Second, the patient population in the current study does differ from those in the UNITI trials. Specifically, when examining the baseline patient characteristics in the UNITI trials, the average duration of disease is 8-12 years, whereas the average duration of disease in the current study’s cohort was ~10-15 years in duration. Furthermore, the baseline CRP levels in the current population were significantly higher compared with those of the population in the UNIT trial, for example, the median CRP level in UNITI was 7.4-10.4 mg/L, while in the current study, the mean CRP level ranged from 16 to 19 mg/mL. Therefore, it is possible that the current study population had more active/longstanding disease which may in turn lead to variable relationships between TDM and endoscopic healing. As demonstrated in the current context, study populations in clinical trials may not perfectly mirror the population seen in clinical practice and this may impact one’s ability to draw conclusions from clinical trial data to real-world patients. Finally, the IM-UNITI trial only examined patients who were on 90-mg Q12/Q8-week dosing, while our study examined patients who were also on an intensified UST dosing (90 mg Q2/4 week). It is plausible that the use of more frequent UST dosing will lead to a higher serum drug levels, which may have an altered association with endoscopic outcomes compared with associations at lower serum drug levels. Ultimately, these are some potential explanations for how the current trial differed from the UNITI trials specifically.

No adverse events were recorded in the current analyses in patients who had higher UST trough levels, while adverse events associated with UST include musculoskeletal pain, dermatologic manifestations, and infection.^26^ There is a sparsity of data examining the association between adverse events and UST trough levels; however, Battat et al. (2017) found that adverse events were not associated with UST trough levels.^15^ Future studies should attempt to identify threshold levels where adverse events may be more likely.

Our study demonstrated no association between serum UST concentrations and endoscopic remission using 2 different measures of this outcome. Limitations of this study include the use of the SES-CD to determine endoscopic response. While the SES-CD is commonly employed both clinically and in research, it is limited in assessing incompletely visualized bowel segments owing to stenosis.^27^ In our study, a small proportion of patients (*n* = 7) had the presence of stenosis/strictures which could have affected endoscopic assessment; however, this likely did not play a significant role in the study outcomes. An additional limitation of the study is that the measuring of UST did not occur at the same time for all patients as there was a relatively wide window of time between endoscopy and serum UST measurement (up to 4 months from endoscopy). While this may have introduced variability in study results, we did not find a difference in outcomes when comparing participants who had serum UST measured within 1 month of endoscopy compared with those who had it measured >1 month. In addition, 86% of the patient population had serum UST levels measured within 1 month of endoscopic assessment; thus, the majority of TDM occurred in close proximity to endoscopy. Future studies may assess the relationship between these outcomes using one time point for serum UST measurement to draw comparisons more easily.

## Conclusions

Our study found no association between endoscopic remission (lack of ulceration or SES-CD < 3) and random serum UST concentrations measured within 4 months of ileo-colonoscopy. Furthermore, there was no threshold serum UST concentration, which predicted endoscopic remission. Future studies should examine serum UST concentration, tissue UST concentration, and their individual predictive value for endoscopic remission. Currently, there are insufficient grounds to advocate for measurement of UST trough levels/optimization of UST dosing to achieve target levels when patients stop responding to maintenance therapy. Moreover, other authors have found that the use of UST TDM has not played a significant role in impacting clinical practice.^28^ As such, further elucidation of the role of UST TDM and its association with treatment endpoints is required before this modality may be employed as standard practice.

## Data Availability

Based upon reasonable request to the corresponding author, the data in this article may be shared. Data are in possession of corresponding authors.
